# Activation of 5-HT_4_ receptors facilitates neurogenesis from transplanted neural stem cells in the anastomotic ileum

**DOI:** 10.1007/s12576-015-0396-1

**Published:** 2015-09-03

**Authors:** Kei Goto, Isao Kawahara, Hiroyuki Inada, Hiromi Misawa, Hiroki Kuniyasu, Junich Nabekura, Miyako Takaki

**Affiliations:** 1 Department of Physiology II, Nara Medical University, School of Medicine, Kashihara, Nara Japan; 2 Department of Molecular Pathology, Nara Medical University, School of Medicine, Kashihara, Nara Japan; 3 Department of Orthopedic Surgery, Nara Medical University, School of Medicine, 840 Shijo-cho, Kashihara, Nara 634-8522 Japan; 4 Division of Homeostatic Development, Department of Developmental Physiology, National Institute for Physiological Sciences, Okazaki, Aichi Japan

**Keywords:** YFP, 5-HT_4_ receptor, Granulation tissue, Myenteric neuron, Neural stem cells

## Abstract

An orally administered serotonin-4 (5-HT_4_) receptor agonist, mosapride citrate (MOS), promotes enteric neurogenesis in anastomoses after gut surgery. We performed gut surgery and transplanted 2 × 10^5^ neural stem cells (NSCs) from the embryonic central nervous system after marking them with the cell linker, PKH26. We found that neurons differentiated from transplanted NSCs (PKH [+]) and newborn enteric neurons differentiated from mobilized (host) NSCs (YFP [+]) in the deep granulation tissue of the anastomotic ileum. MOS significantly increased the number of PKH (+) and YFP (+) neurons by 2.5-fold (*P* < 0.005) (*n* = 4). The distribution patterns of PKH (+) neurons and YFP (+) neurons were similar along the depth of the anastomosis. A 5-HT_4_ receptor antagonist, SB-207266, abolished these effects of MOS (*n* = 4). Our results indicate that neurogenesis from transplanted NSCs is potentiated by activation of 5-HT_4_ receptors. Thus, a combination of drug administration and cell transplantation could be more beneficial than cell transplantation alone in treating Hirschsprung’s disease and related disorders.

## Introduction

We have previously shown that enteric neural 5-HT_4_ receptors activated by mosapride citrate (MOS) promote reconstruction of the impaired enteric neural circuit after transection and formation of an anastomosis. This treatment also promoted recovery of the “defecating reflex” [1, 2] in the rectum of guinea pigs [3]. This plasticity of enteric neurons is brought about by mobilized neural stem cells (NSCs) [3]. Furthermore, we established the differentiation of gut-like organs from mouse embryonic stem cells (ES gut) and showed that MOS treatment enhanced neural network formation in these organs [4]. Liu and Gershon [5] reported that other 5-HT_4_ receptor agonists increased the number and neurite length of enteric neurons developed in vitro from neural crest-derived precursors. Furthermore, Liu et al. [6] demonstrated that neuroprotection and/or neurogenesis in the adult mouse enteric nervous system (ENS) is mediated by 5-HT_4_ receptor.

Recently, we investigated whether MOS could promote enteric neurogenesis at anastomotic sites in the murine ileum in vivo [7]. Newborn neurons are typically distributed within the thick granulation tissues. The granulation tissues are newly formed connective tissues composed of fibroblasts and blood capillaries after the transection and anastomosis of the rectum [1]. Traditional fluorescence microscopy including confocal microscopy is unsuitable for high-resolution deep imaging of the 300–400-µm-thick granulation tissue, as previously reported [7]. Imaging of newly formed neurons and axons is severely limited even in in vitro whole-mount preparations containing the longitudinal muscle, myenteric plexus, and circular muscle layers. Two-photon-excited fluorescence microscopy (2PM) overcomes this limitation by providing enhanced optical penetration. 2PM can be used to perform cellular imaging several hundred microns deep in the native environment [8]. We previously confirmed the expression of GFP in the cytoplasm of enteric neurons of the ileum of Thy1-GFP mice [9]. Using 2PM and Thy1-GFP mice, we obtained three-dimensional in vivo images of reconstructed enteric neural circuits within the thick tissue in the ileum in their native environment [7]. Another method using confocal laser endomicroscopy with a probe for in vivo imaging of the proper muscle and myenteric neurons in pig models has also been reported [10].

As demonstrated in the rectum [3], the study in the ileum revealed that MOS-activated neural 5-HT_4_ receptors facilitates neurogenesis from mobilized NSCs [7]. In the present study, we hypothesized that NSCs from the hippocampus and subventricular zone (SVZ) of mouse embryos could be used for transplantation to achieve neurogenesis in the gut [11]. Using 2PM the present study aimed to determine whether activation of 5-HT_4_ receptors by MOS promotes neurogenesis from transplanted NSCs in their native environment after gut surgery in Thy1 promoter YFP mice.

## Materials and methods

### Cell culture and neural stem cells (NSCs)

To confirm their quality, NSCs (catalog no. F-MUBNF-0101, Cyagen Biosciences) from the hippocampus and SVZ of day-12.5 post-coitus C57BL/6 mouse embryos supplied in a cryovial containing 1 × 10^6^ cells were seeded on a gelatin-coated dish and then cultured as neurospheres in neural stem cell growth medium (catalog no. F-GUXNX-9011, Cyagen Biosciences) for 4 days. Microscopic photographs were taken under an inverted microscope (Nikon Ti-S100, Tokyo, Japan).

### Drugs

Brain-derived neurotrophic factor (BDNF 10 ng ml^−1^; Upstate, Lake Placid, NY), mosapride citrate (MOS 1 µM; generous gift from Dainippon-Sumitomo Pharmaceutical Co., Ltd, Osaka, Japan), and GR (GR113808 10 µM; Wako Pure Chemical Industries, Osaka, Japan) were added during each cell culture.

### Cell membrane labeling by PKH26 yellow-orange cell linker kits

PKH26 (Sigma-Aldrich, St. Louis, MO) is chosen to be the cell linker dye for in vivo cell-tracking studies [12], especially when labeled cells are followed for longer than a few weeks [13], because it does not inhibit stem cell proliferation and produces no toxicity to the stem cells [14]. Applications of dye dilution proliferation analysis include identification of quiescent or slowly dividing stem or progenitor cells in newly formed tissues [14], such as the granulation tissue at the anastomosis. PKH26 could label these stem or progenitor cells [14]. For this reason, we used PKH26 as a cell tracer.

NSCs (catalog no. F-MUBNF-0101, Cyagen Biosciences) from the hippocampus and SVZ from day-12.5 post-coitus C57BL/6 mouse embryos were supplied in a cryovial containing 1 × 10^6^ cells. The NSC suspension was transferred to a 15-ml conical tube. Ten milliliters serum-free medium was added to the conical tube and mixed well. After centrifugation at 400×*g*, 5 min, the supernatant was carefully removed by aspiration, and the remaining cell pellet was resuspended with gentle pipetting in 1 ml of Diluent C. The 1 ml of dye solution (4 µl of the PKH26 in the 1 ml of Diluent C) was quickly added to 1 ml of the cell suspension, and the cell/dye suspension was mixed by immediate pipetting and finally incubated for 5 min at 25 °C. The labeling was stopped by 2 ml of 1 % BSA, and the cell/dye suspension was reincubated for 1 min and centrifuged at 400×*g*, 10 min at 25 °C, after which the supernatant was carefully removed. The remaining cell pellet resuspended in 10 ml of complete medium was centrifuged at 400×*g*, 5 min, at 25 °C and washed with 10 ml of complete medium. We repeated these procedures twice to ensure removal of unbound dye. After the final wash, the cell pellet was resuspended in 1 ml of PBS. The final cell concentration was not altered. The labeled cell suspension (0.2 ml; 2 × 10^5^) was injected into the tail vein for cell transplantation in each mouse.

### Description and preparation of transgenic mice

All other studies mentioned below were carried out in strict accordance with the recommendations in the Guide for the Care and Use of Laboratory Animals of the National Institutes of Health. All relevant experimental protocols were approved by the Ethics Review Committee for Animal Experimentation of the National Institute for Physiological Sciences (permission no. 11A114). All surgical procedures were performed under Nembutal anesthesia, and all efforts to minimize suffering were made.

We used a transgenic mouse, Thy1 promoter YFP mouse, H-line. At first, we confirmed the expression of cytoplasmic YFP in enteric neurons. The abdomen of transgenic mice (8–12 weeks of age) was opened under anesthesia with Nembutal (50 mg kg^−1^ IP) by a lower midline laparotomy [7]. This approach avoided the disturbance of blood flow and extrinsic innervation via the mesenteric nerves. The ileum was transected (5–6 cm oral from the ileocecal sphincter), and an end-to-end one-layer anastomosis was performed at 36–37 °C, as previously reported [7]. Immediately after surgery, NSCs labeled with PKH26 were transplanted through the tail vein. After cell transplantation, mice daily drank 0.1 % DMSO solution (vehicle) (*n* = 4), MOS (100 µM) in vehicle (*n* = 8), or a selective 5-HT_4_-antagonist for oral administration [SB-207266 (SB 10–50 µM)] [15] plus MOS (100 µM) in vehicle (*n* = 4) for 2 weeks (with fasting for the first 2 days, then feeding ad libitum for the final 12 days).

### In vivo two-photon excitation microscopy

Fourteen days after the cell transplantation, the abdomen was opened by a lower midline laparotomy under anesthesia with nembutal (50 mg kg^−1^) at 36–37 °C, and the surgical site of the ileum was fixed into the chamber for 2PM, avoiding disturbance of the blood supply [7]. Additional nembutal was administered as required. We suppressed spontaneous ileum motilities for microscopy by pinning down the preparation and by intraluminal injection of a smooth muscle relaxant, papaverine (1 mM; 0.1–0.2 ml). The imaging rate was 2.711 s frame^−1^. The 2PM (FV1000-MPE, Olympus, Tokyo) used for in vivo imaging of YFP-labeled structures was customized using a water-immersion objective lens (40×, NA0.8, Olympus) at a zoom of 1.0. A Ti-sapphire laser (MaiTai Hp, Spectral Physics, Mountain View, CA, USA) was tuned to the excitation wavelength for YFP (950 nm) (yellowish green fluorescence), as previously reported [7]. Because of a broadening excitation wavelength (blue shift), this excitation wavelength can excite PKH26 (excitation maximum 551 nm and emission maximum 567 nm) (yellow-orange fluorescence). The stacked image with *z* axis consisted of 200 optical sections taken 1 µm apart (within 400 µm from the serosal surface). Counting of neurons was done in each optical section of each of nine fields, where we could differentiate two types of neurons as orange (PKH26+) and yellow (YFP+). Throughout imaging the photomultiplier tube setting and excitation power (~50 mW) were kept constant. Some fluorescence images taken by a 2PM were analyzed using Image J (1.48v, NIH, Bethesda, MD, USA), and some three-dimensional images were made by IMARIS (Bitplane, South Windsor, CT, USA).

### Fluorescence imaging by confocal microscopy and immunohistochemistry of sectioned preparations

Mice were euthanized by an excessive dose of nembutal after in vivo imaging was finished. Fixed frozen blocks and sections of mouse tissues for immunohistochemistry (IHC) and fluorescence imaging by confocal microscopy were obtained from Genostaff Co., Ltd (Tokyo, Japan). The ileum along with an anastomosis was fixed with 4 % paraformaldehyde at 4 °C for 16 h and was embedded in Cryo Mount 1 (MUTO Pure Chemicals, Co., Ltd., Tokyo, Japan) according to the proprietary procedures. From each block 6-µm sections were consecutively cut. Each section was examined with a confocal microscope (Olympus FV1000, Tokyo, Japan). For IHC, each section was washed with PBS to remove the excess compound. Antigen was retrieved by heat treatment at 80 °C, 40 min, with sodium citrate buffer at pH 6.0. Endogenous peroxidase blockade with 0.3 % H_2_O_2_-methanol, 30 min, was performed, followed by incubation with Protein Block (DAKO Corp., Carpinteria, CA, USA) and avidin/biotin blocking kit (Vector Laboratories, Inc., CA, USA). The sections incubated with mouse monoclonal antibody for PGP9.5 (catalog no. ab8189, 0.4 µg ml^−1^, Abcam PLC., Cambridge, UK) at 4 °C overnight were then incubated with biotin-conjugated goat anti-rabbit Ig (DAKO) diluted 1:600, 30 min, at room temperature and followed by the addition of peroxidase conjugated streptavidin (Nichirei Biosciences Inc., Tokyo, Japan), 5 min. Diaminobenzidine solution (DAKO) visualized peroxidase activity. The sections counterstained with Mayer’s hematoxylin (MUTO) were dehydrated and then mounted with Malinol (MUTO).

### Statistics

Multiple comparisons by one-way analysis of variance (ANOVA) with post hoc Bonferroni’s test were performed. A value of *P* < 0.05 was considered statistically significant. All data are expressed as the mean ± standard deviation (SD).

## Results

### Effects of MOS on NSCs after 4 days in culture

**Fig. 1:**
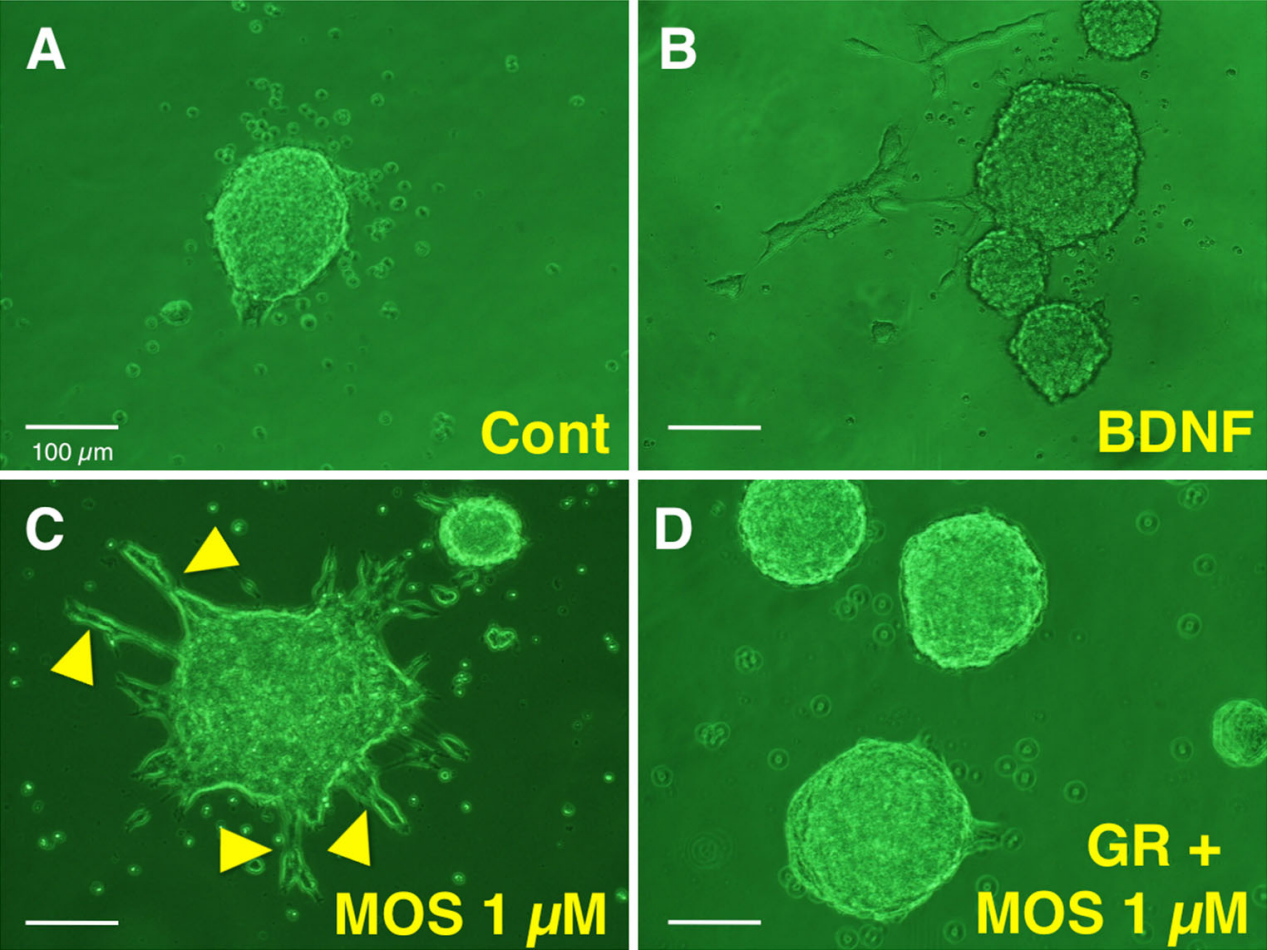
Effects of brain-derived neurotrophic factor (BDNF), mosapride citrate (MOS), and MOS plus GR113808 (GR) on neurospheres formed by neural stem cells from the embryonic hippocampus and subventricular zone after 4 days of culture in neural stem cell growth medium. **a** Control without treatment. **b** Treatment with 10 ng ml^−1^ BDNF. **c** Treatment with 1 µM MOS. **d** Treatment with 1 µM MOS and 10 µM GR. *Arrowheads* indicate projections. *Cal bar* 100 µm

As the preliminary for cell transplantation, we examined the effects of MOS on NSCs in culture. NSCs formed neurospheres after 4 days of culture in NSC growth medium in controls. BDNF facilitated the formation of enteric neural networks in ES guts, but GDNF did not [16]. We therefore examined the effects of BDNF on NSCs in culture. BDNF (10 ng ml^−1^) weakly facilitated the outgrowth of projections from neurospheres, and MOS (1 µM) more potently facilitated the outgrowth of projections from neurospheres, but GR (10 µM), a selective 5-HT_4_ receptor antagonist, abolished MOS-induced effects on neurospheres (Fig. 1). From these results, we judged these NSCs could be used for the following cell transplantation.

### In vivo images of the anastomotic region in MOS-treated YFP mice 2 weeks after NSC transplant

**Fig. 2:**
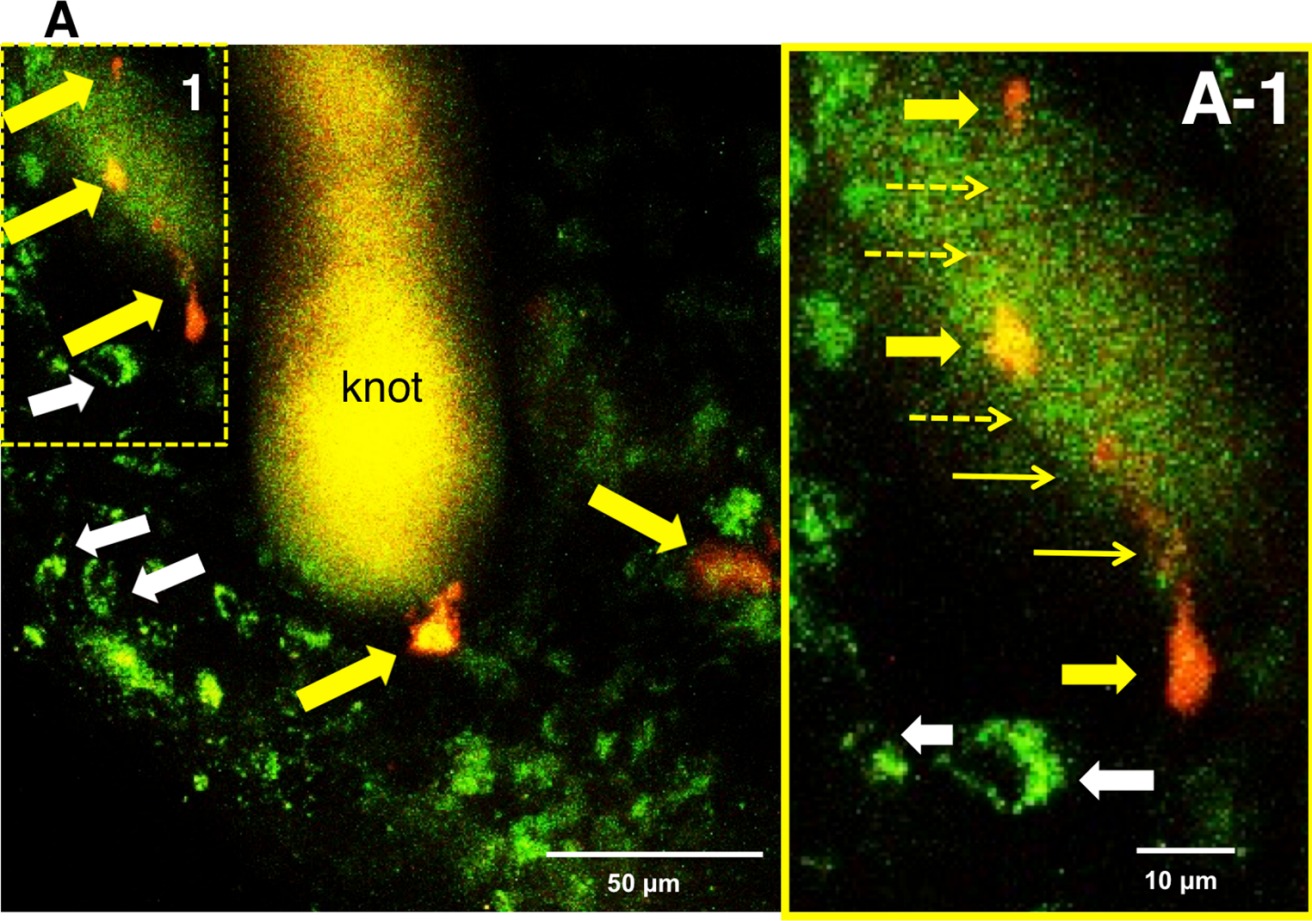
Two photon-excited fluorescence microscopy (Two-PM) in vivo images of the anastomotic region in an NSC-transplanted and MOS-treated YFP mouse for 2 weeks. Around the knot at the anastomotic site we obtained an image including newborn neurons differentiated from mobilized (host) NSCs (yellowish *green fluorescence*) and neurons differentiated from transplanted NSCs (*yellow-orange* *fluorescence*). **a** Stacked images with *Z* axis to a total depth of 1–201 µm. *Yellow arrows* indicate nerve cells differentiated from transplanted NSCs. *White arrows* indicate newborn neurons from mobilized NSCs. The area framed by a dotted square in (**a**) was enlarged into (**a-1**). **a-1** *Thick yellow* *arrows* indicate neurons from transplanted NSCs. *Thin yellow arrows* indicate cell processes connecting neurons, and the *dotted yellow arrows* indicate presumed cell processes. *White arrows* indicate newborn neurons from mobilized NSCs

Initially we confirmed that enteric neurons were visible in vivo in the terminal ileum, which had a maximal thickness of 50 µm at the myenteric ganglion level in an intact Thy1-YFP mouse. This has been previously reported for Thy1-GFP mice [7]. In Thy1-YFP mice treated with MOS (100 µM), 2 weeks after gut surgery and NSC transplantation, yellowish-green fluorescence-positive (YFP [+]) neurons that possibly differentiated from the mobilized (host) NSCs were observed around the knot at the anastomotic site in the thick granulation tissue at a depth of 1–201 µm from the serosal surface. Surprisingly yellow-orange PKH26 fluorescence-positive (PKH [+]) neurons that possibly differentiated from the transplanted NSCs were also observed in the same site (Fig. 2a, a-1) in three out of four mice. In one mouse, we could not observe the anastomotic site because of its severe adhesion. As shown in Fig. 2a-1, it seems likely that newly differentiated neurons project their axons to other neurons.

### Confocal microscopy images of the longitudinal ileum section including anastomosis

**Fig. 3:**
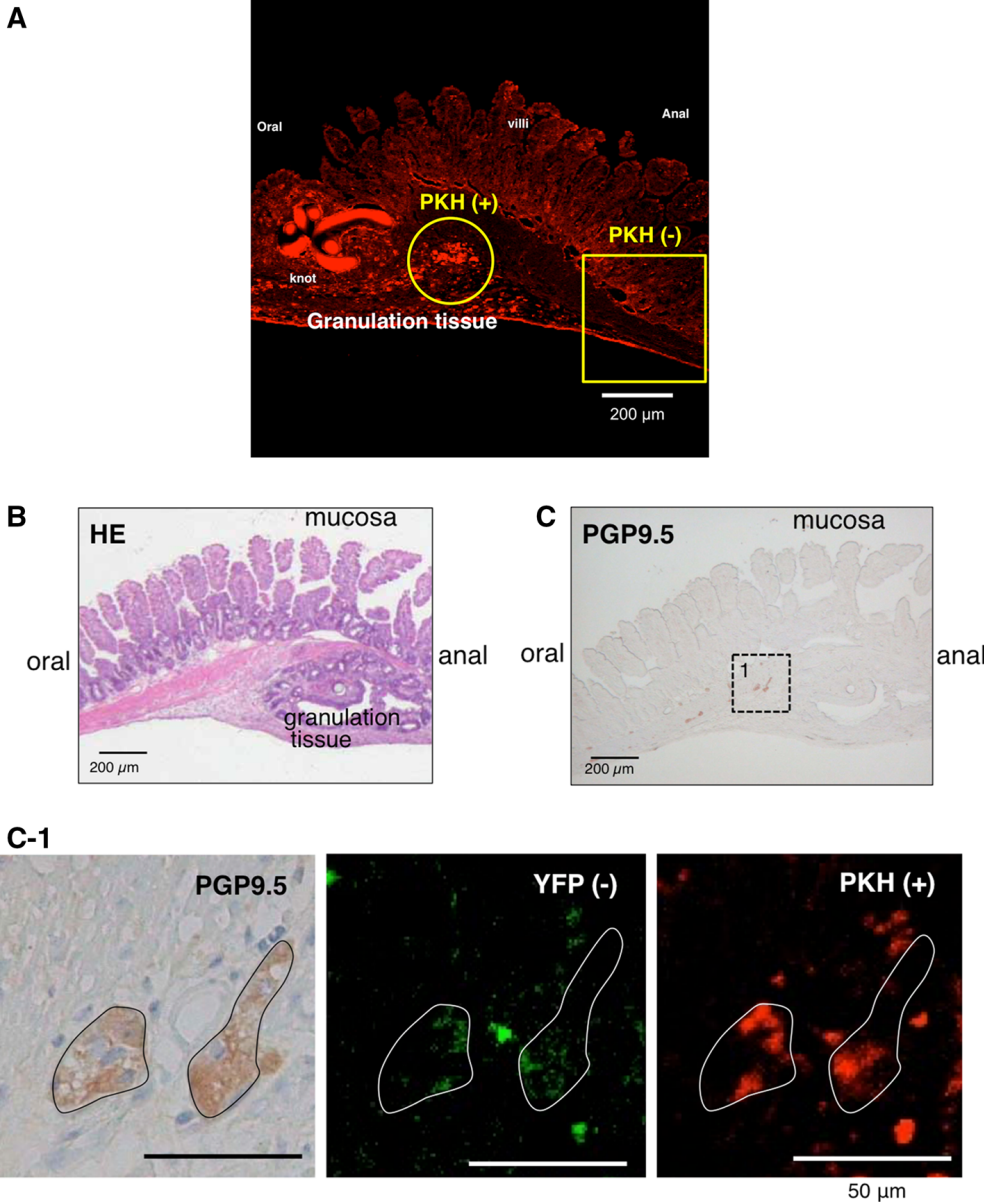
Confocal microscopy images of the longitudinal ileum section including the anastomosis in a MOS-treated YFP mouse 2 weeks after NSC transplant. **a** PKH26 (+) aggregates were found only in the granulation tissue at the anastomosis and not found outside the granulation tissue. **b** Hematoxylin-eosin staining. **c** Anti-PGP9.5 antibody immunostaining. *Left side* Oral side of the gut. **c-1** *Enlarged figures* correspond to a square 1 in (**c**) in the granulation tissue. DAB products indicate PGP9.5 (+) ganglionic cells. PKH26 (+) cells were included in PGP9.5 (+) ganglia but YFP (+) cells were not included

After in vivo imaging with 2PM, the longitudinal ileum section including the anastomosis was observed under a confocal microscope in MOS-treated mice (*n* = 4). PKH26 (red fluorescence) (+) aggregates were found at the border of the granulation tissue but not outside the granulation tissue (Fig. 3a). These aggregates of the transplanted NSCs seem to be mobilized to the granulation tissue.

### Immunohistochemistry (IHC) of the longitudinal ileum section including the anastomosis with the anti-PGP9.5 antibody

To determine whether the PKH26 (+) aggregates were differentiated neurons from the transplanted NSCs, the immunoreactivity to anti-PGP9.5 antibody was examined (*n* = 4). PGP9.5-immunopositive (+) cells were expectedly observed in the granulation tissue (Fig. 3b, c), some of which formed neural ganglia (Fig. 3c). The same section showed both PGP9.5 immunopositivity and PKH fluorescence. PKH26 (+) and PGP9.5 (+) cells were neurons differentiated from the transplanted NSCs derived from the embryonic hippocampus and SVZ (Fig. 3c-1). PKH26 (+) cells clustered within two PGP9.5 (+) ganglia, indicating that the clustered transplanted NSCs formed two neural ganglia.

### Quantitative analysis of new neurons differentiated from transplanted and host NSCs at the anastomotic region in MOS-treated YFP mice after 2 weeks

**Fig. 4:**
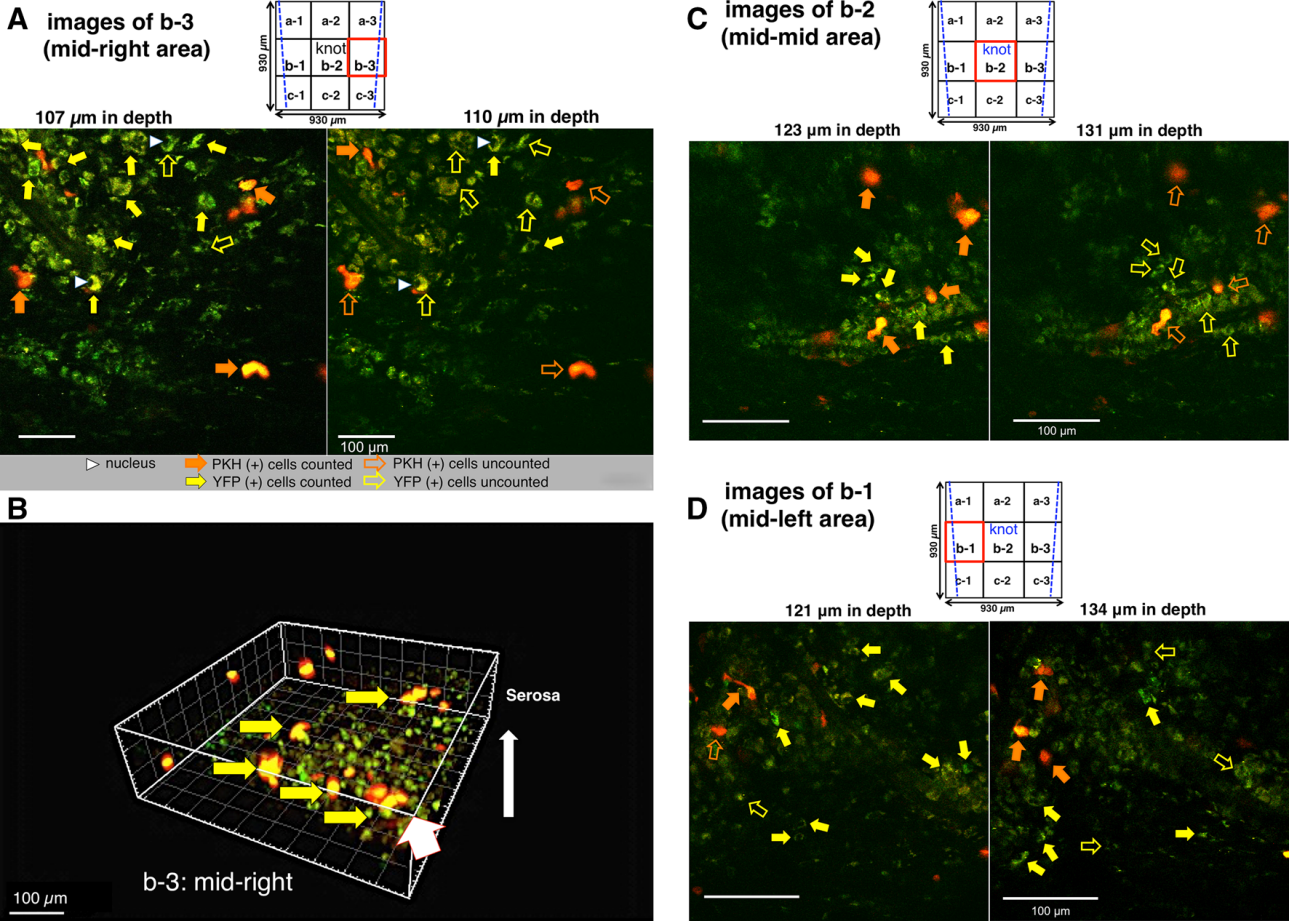
Two-PM images of the anastomotic region in an NSC-transplanted and MOS-treated YFP mouse for 2 weeks. PKH26 fluorescence (+)/YFP fluorescence (+) [PKH26 (+)/YFP (+)] neurons distributed in each of the three areas (b-1, -2, -3) of each of nine field (a-1–c-3; field size: 310 µm × 310 µm) around the knot at the anastomosis were demonstrated. *Two blue lines* (*upper square*) indicate both ends of the anastomosis. **a** Mid-right areas (b-3) were demonstrated at depths of 107 and 110 µm. *Each white arrowhead* indicated nucleus. **b** A mid-right area (b-3) was demonstrated three dimensionally from a direction shown by a *thick white arrow*. *Yellow arrows* indicate clustered PKH26 (+) neurons. **c** Mid-mid areas (b-2; knot area) were demonstrated at depths of 123 and 131 µm. **d** Mid-left areas (b-1) were demonstrated at depths of 121 and 134 µm. In (**a**, **c**, **d**), *orange solid arrows* indicated PKH26 (+) neurons, and *yellow solid arrows* indicated YFP (+) neurons (not all). Overlapped neurons between both depths were not counted, as indicated by each *open arrow*

PKH26 (+) neurons (shown by solid orange arrows) or YFP (+) neurons (shown by solid yellow arrows) could be correctly counted in examples of the mid-right area (b-3) at depths of 107 and 110 µm from the serosal surface (Fig. 4a). Examples of the mid-right area (b-3) are shown three dimensionally in Fig. 4b. It was clearly observed that PKH26 (+) neurons clustered together in new ganglionic structures. In addition, PKH26 (+) neurons or YFP (+) neurons could be correctly counted in examples of the mid–mid area (b-2; knot area) at depths of 123 and 131 µm (Fig. 4c) and of the mid-left area (b-1) at a depth of 121 and 134 µm from the serosal surface (Fig. 4d). Around the knot of thread at the anastomosis, 59 PKH (+) neurons and 693 YFP (+) neurons were counted in nine images × 140 optical sections (=with a total *z* axis depth of 140 µm). The other three mice treated with MOS also showed similar results.

### Quantitative analysis of new neurons differentiated from transplanted and host NSCs at anastomotic region in SB + MOS-treated YFP mice after 2 weeks

**Fig. 5:**
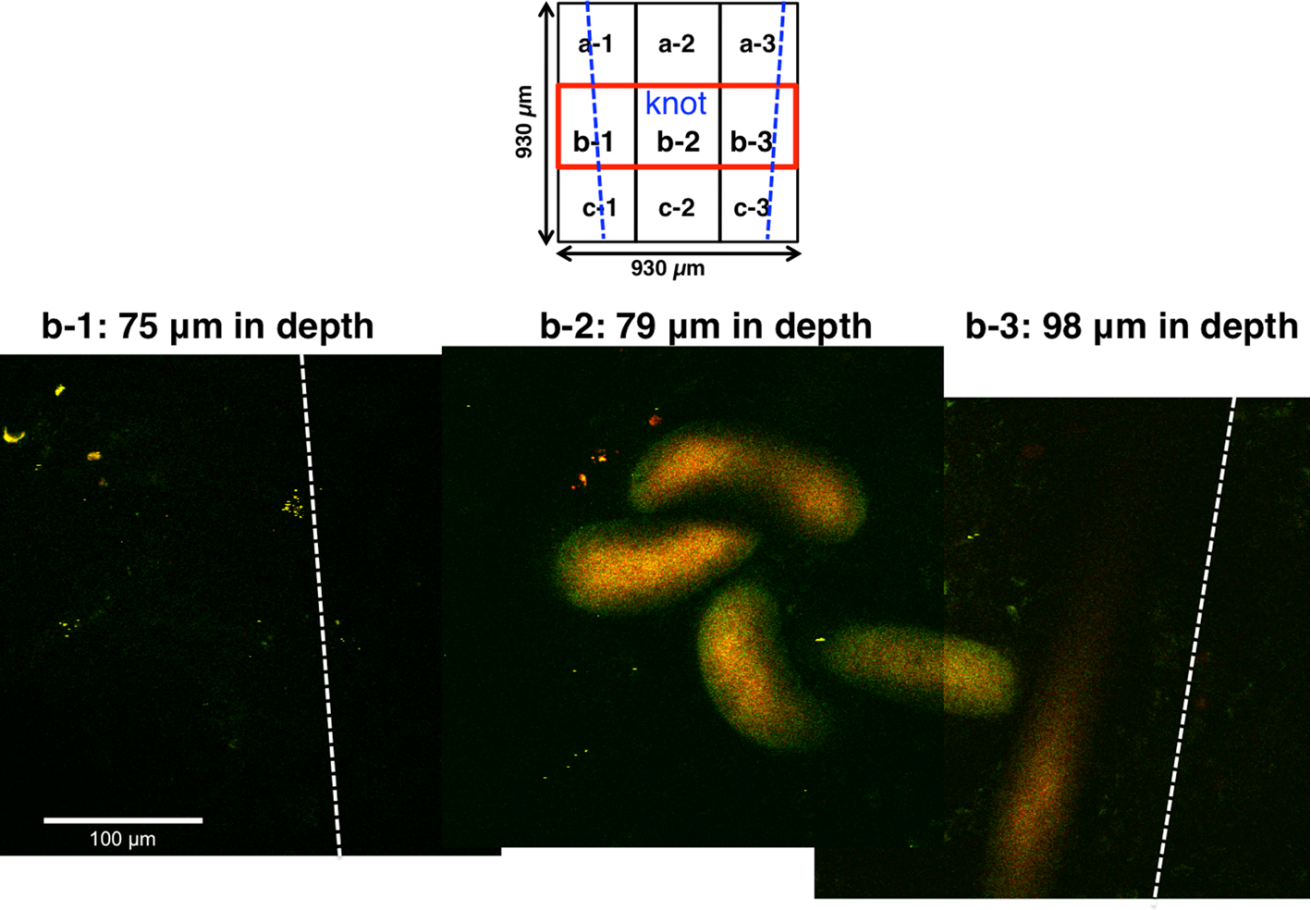
Two-PM images of the anastomotic region in SB-207266 (SB) and MOS-treated YFP mouse 2 weeks after NSC transplant. Each image of PKH26 (+)/YFP (+) neurons distributed in three (b-1, -2, -3) of each of nine fields (a-1–c-3; field size: 310 µm × 310 µm) around the knot at the anastomosis was demonstrated. Each pair of *blue* (*upper square*) or *white dotted lines* (*lower images*) indicates both ends of the anastomosis. PKH26 (+)/YFP (+) neurons in b-1 at a depth of 75 µm, b-2 at a depth of 79 µm and b-3 at a depth of 98 µm were hardly visible

In mice treated with the 5-HT_4_ antagonist, SB 207266 (SB), and MOS, 2 weeks after gut surgery and NSC transplantation, we observed each image from nine visual fields around the knot. PKH26 (+) neurons or YFP (+) neurons could be correctly counted in examples of the mid-left, mid–mid, and mid-right area (b-1, -2, -3) at depth of 75, 79, and 98 µm from the serosal surface (Fig. 5). Twenty-six PKH (+) neurons and 136 YFP (+) neurons were counted in nine images × 120 optical sections (=a total *z* axis depth of 120 µm). Thus, MOS-induced facilitation of neurogenesis in both host and transplanted NSCs was largely inhibited by the concomitantly administered 5-HT_4_ receptor antagonist, SB. The other three mice treated with MOS and SB showed similar results.

**Fig. 6:**
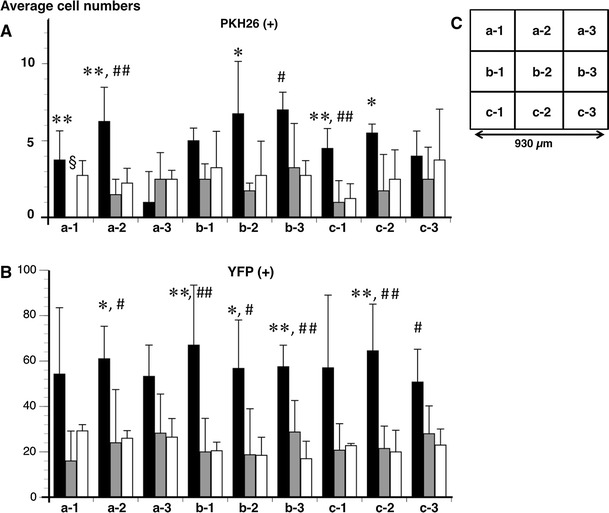
The average numbers of PKH26 (+) (**a**) and YFP (+) (**b**) neurons in each of nine fields at the anastomotic region. The average numbers of both neurons were compared among the MOS (*n* = 4), SB + MOS (*n* = 4), and vehicle-treated (*n* = 4) groups. Each name of the nine fields was demonstrated in (**c**). *Black bar* MOS; *gray bar* SB + MOS; *white bar* vehicle. *P < 0.05 MOS versus SB + MOS. **P < 0.005 MOS versus SB + MOS. ^#^ *P* < 0.05 MOS versus vehicle. ^##^ *P* < 0.005 MOS versus vehicle. ^§^ *P* < 0.05 SB + MOS versus vehicle

The average cell numbers of PKH26 (+) and YFP (+) neurons in the vehicle were not significantly different among each of the nine fields (from a-1 to c-3) (Fig. 6). The facilitating effect of neurogenesis by MOS on PKH26 (+) neurons was significant in a-2, b-3, and c-1 (*P* < 0.05 or *P* < 0.005) (Fig. 6a). The facilitating effect of neurogenesis by MOS on YFP (+) neurons was significant in a-2, b-1–3, and c-2–3 (*P* < 0.05 or *P* < 0.005) (Fig. 6b). The antagonizing effect of neurogenesis by SB on PKH26 (+) neurons was significant in a-1–2, b-2, and c-1–2 (*P* < 0.05 or *P* < 0.005) (Fig. 6a). The antagonizing effect of neurogenesis by SB on YFP (+) neurons was significant in a-2, b-1–3, and c-2 (*P* < 0.05 or *P* < 0.005) (Fig. 6b).

**Fig. 7:**
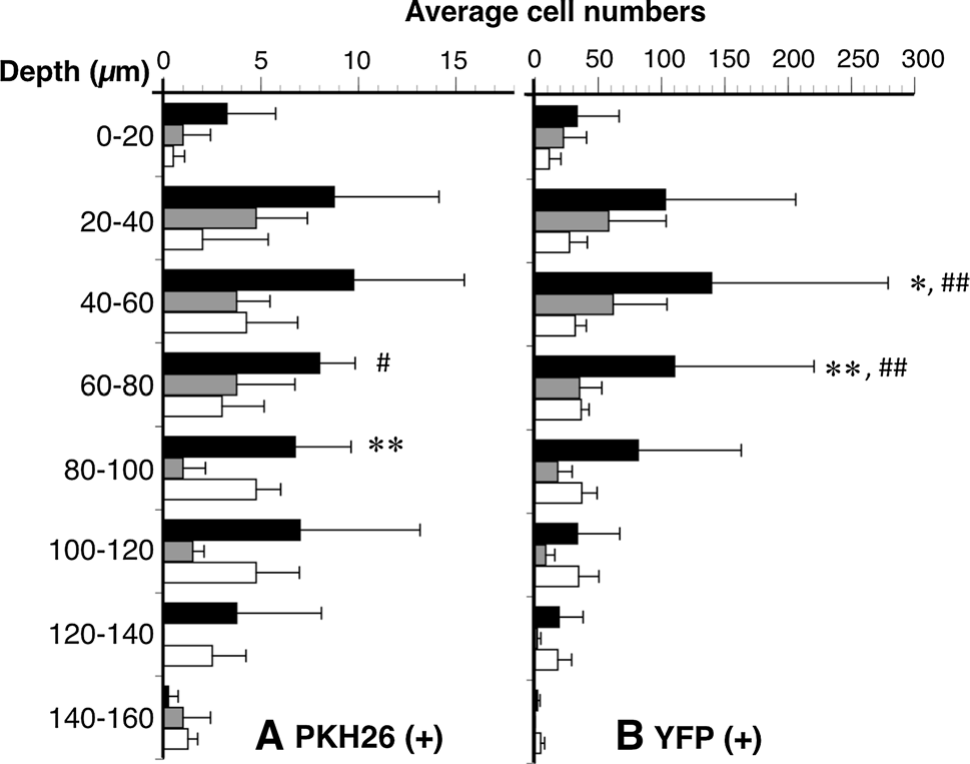
The average numbers of PKH26 (+) (**a**) and YFP (+) (**b**) neurons within depths of 0–160 µm at every 20 µm. The average numbers of both neurons were compared among MOS (*n* = 4), SB + MOS (*n* = 4), and vehicle-treated (*n* = 4) groups. *Black bar* MOS; *gray bar* SB + MOS; *white bar* vehicle. *P < 0.05 MOS versus SB + MOS. **P < 0.005 MOS versus SB + MOS. ^#^ *P* < 0.05 MOS versus vehicle. ^##^ *P* < 0.005 MOS versus vehicle

PKH26 (+) or YFP (+) neurons in vehicle distributed at a depth of 0–160 µm (Fig. 7). The facilitating effect of MOS on neurogenesis in PKH26 (+) neurons was significant at a depth of 60–80 µm (*P* < 0.05; Fig. 7a). The antagonizing effect of SB on neurogenesis in PKH26 (+) neurons was significant at a depth of 80–100 µm (*P* < 0.005; Fig. 7a). The facilitating effect of MOS on neurogenesis in YFP (+) neurons was significant at a depth of 40–80 µm (*P* < 0.005; Fig. 7b). The antagonizing effect of SB on neurogenesis in YFP (+) neurons was significant at a depth of 40–80 µm (*P* < 0.005; Fig. 7b).

**Fig. 8:**
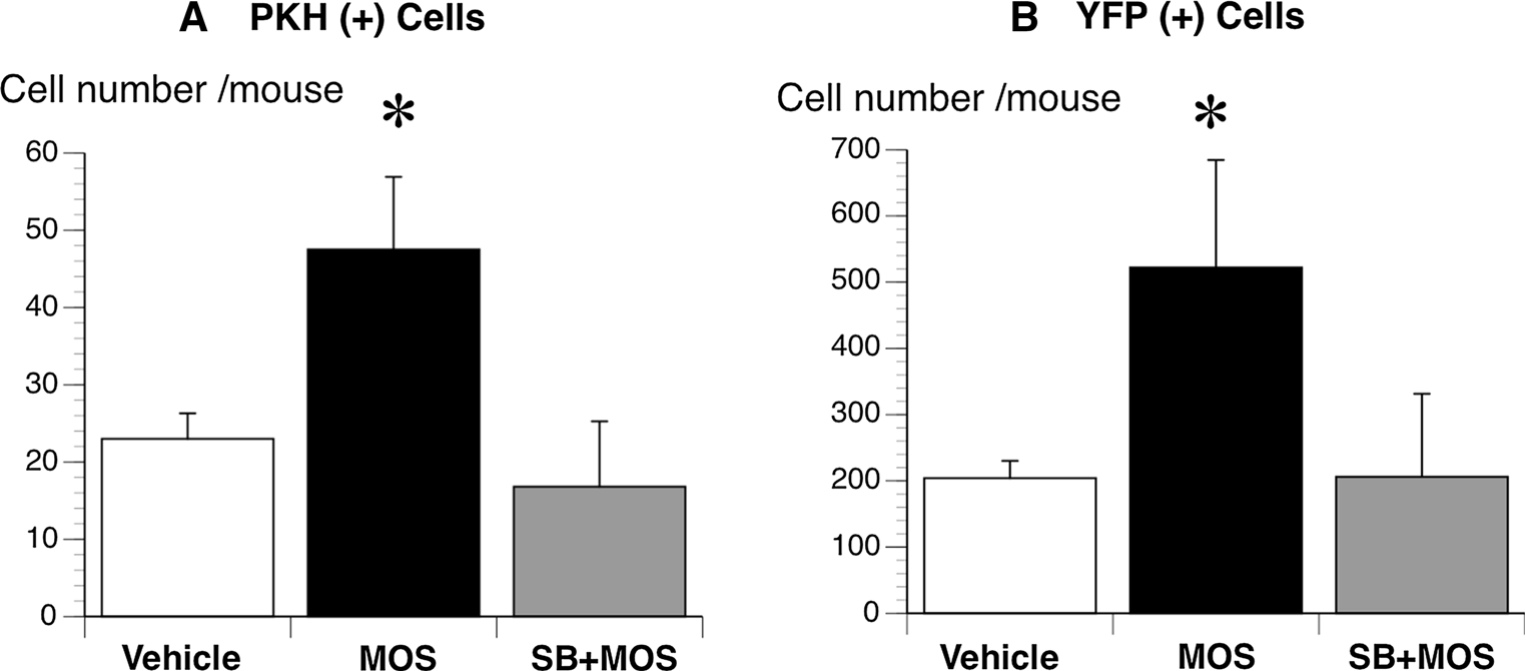
The average numbers of PKH26 (+) (**a**) and YFP (+) (**b**) neurons in all nine visual fields at the anastomosis. The average numbers of both neurons were compared among the MOS (*n* = 4), SB + MOS (*n* = 4), and vehicle-treated (*n* = 4) groups. *Black bar* MOS; *gray bar* SB + MOS; *white bar* vehicle. *P < 0.005 MOS versus SB + MOS and versus vehicle

The total number of PKH26 (+) neurons was one-tenth of that of YFP (+) neurons; MOS significantly increased the total number of PKH26 (+) neurons to 2.5-fold (48 ± 9 per 0.8649 mm^2^; *P* < 0.005) and that of YFP (+) neurons to a similar 2.5-fold (522 ± 163 per 0.8649 mm^2^; *P* < 0.005); SB completely antagonized these effects of MOS on PKH26 (+) (17 ± 8 per 0.8649 mm^2^; *P* < 0.005) and YFP (+) neurons (206 ± 126 per 0.8649 mm^2^; *P* < 0.005) (Fig. 8).

## Discussion

As mentioned in our previous study [7], it is impossible for traditional fluorescence microscopy to visualize neurons within thick tissue because of the impermeability of the laser into the tissue. Therefore, 2PM was employed for three-dimensional neural imaging within the thick tissue formed after gut surgery in the ileum of Thy1-GFP mice [7].

This is the second study employing in vivo enteric neural imaging with 2PM, although in vivo enteric neural imaging with less invasive confocal laser endomicroscopy has been recently reported [10]. We detected newly generated neurons derived from two different (transplant and host) sources of NSCs in the thick tissue at the anastomosis; almost all neurons were distributed within 160 µm. In the present study, neurons that differentiated from the transplanted PKH-labeled NSCs derived from the embryonic hippocampus and SVZ were found, as reported in the CNS [17] and in a genetic mouse model of gastroparesis [11]. Enteric neurons expressing cytoplasmic YFP derived from host NSCs were also found (Fig. 2).

One-week treatment with MOS after surgical anastomosis facilitated neurogenesis of enteric neurons including progenitors in the granulation tissue; however, the vehicle (DMSO) also induced about one-fourth of the neurogenesis facilitated by MOS [7]. This indicates that the formation of a small number of enteric neurons after anastomosis is possible without cell transplantation. In the present study, we found a significant increase in the number of neurons differentiated from the transplanted and host NSCs in the granulation tissue of MOS-treated mice 2 weeks after surgery. The transplanted NSCs (PKH+ cells) and host NSCs were mobilized to the anastomosed area and/or survived, proliferated, and finally differentiated into neurons [11, 18, 19]. MOS clearly accelerated these processes after surgery. We speculated that these processes are probably initially activated by the release of chemokines from the anastomosis, although further studies are needed. The effects of MOS on neurogenesis from the transplanted and host NSCs were completely antagonized by the 5-HT_4_ receptor antagonist, which indicates that MOS facilitates the formation of neurons from the transplanted and host NSCs via 5-HT_4_ receptor activation.

In the present study, the PKH (+) neurons were found not only in shallow but also in deep granulation tissue. This distribution pattern of PKH (+) neurons, along with the depth of the granulation tissue, was similar to that of YFP (+) newborn enteric neurons that differentiated from host NSCs. These results suggest that host NSCs from the neural crest [7] and transplant NSCs migrated from the outside into the deep granulation tissue by blood flow, as in the colonic ENS, which is developed by a population of trans-mesenteric enteric neural crest cells [20].

Hirschsprung’s disease (HSCR) and related diseases occur because of failure in the development of ENS. HSCR pathogenesis is caused by mutations in genes encoding the Ret receptor tyrosine kinase (RET) and endothelin receptor type B [18, 21]. We have previously reported that MOS increases the mRNA level of RET in the cells mobilized into an implanted gel sponge in a rat subcutaneous model (not a gut model); this increase in RET mRNA was completely blocked by treatment with a 5-HT_4_ receptor antagonist [19]. RET seems to be the target molecule of MOS, and RET inhibitors suppress MOS-induced neurogenesis [22].

Recently, Findlay et al. [23] reported that the brain-derived progenitors isolated from the cerebral cortex are less efficient than enteric neural progenitors in the colon. In their report, neurons from ENS progenitors clustered together in the ganglia, whereas neurons from CNS progenitors were mostly solitary. However, in the present study the transplanted NSCs derived from the hippocampus and SVZ clustered together in the ganglia (see Figs. 3c-1, 4b). Thus, a combination of drug administration and cell transplantation could be more beneficial than cell transplantation alone in treating HSCR and related disorders [24].

In conclusion, 2PM can enable high-resolution deep imaging of thick tissue at the anastomotic site in the small intestine in vivo, including visualization of newly formed network structures differentiating from two different (host and transplant) NSCs. Our results demonstrate that orally administered MOS enhances neurogenesis from transplant NSCs in addition to that from host NSCs by activation of enteric neural 5-HT_4_ receptors. In the future, we expect to perform functional studies of new network structures over the thick granulation tissue at the anastomosis using in vivo imaging with 2PM and transgenic mice expressing genetically encoded calcium indicators such as GCaMPs [25].
